# A systematic review of the barriers, enablers and strategies to embedding translational research within the public hospital system focusing on nursing and allied health professions

**DOI:** 10.1371/journal.pone.0281819

**Published:** 2023-02-16

**Authors:** Sophie Smith, George Johnson

**Affiliations:** 1 Sydney School of Public Health, University of Sydney, Sydney, NSW, Australia; 2 Sydney Institute for Women, Children and their Families, Sydney Local Health District & Sydney School of Public Health, University of Sydney, Sydney, NSW, Australia; Institute of Advanced Materials, IAAM, SWEDEN

## Abstract

**Aim:**

This systematic review aims to investigate, identify, and compare evidence related to the barriers, enablers, and strategies to embedding translational research within a public hospital system focussed on nursing and allied health disciplines.

**Methods:**

A systematic review looking at the international literature on the barriers, enablers and strategies in embedding translational research within a public health system addressing nursing and allied health professions. The study channelled the PRISMA reporting guidelines for systematic reviews and meta-analyses. Databases searched were Medline, Embase, Scopus and Pubmed from January 2011 to December 2021 (inclusive). A quality assessment was conducted of literature using the mixed methods appraisal tool 2011 version.

**Results:**

Thirteen papers met the inclusion criteria. The studies included were from Australia, Saudi Arabia, China, Denmark and Canada. Occupational therapy and physiotherapy were the only two allied health disciplines identified in the search process. The review found considerable inter-relationships between the enablers, barriers, and strategies to embedding research translation in a public hospital setting. Three over-arching themes ‘leadership, organisational culture and capabilities’ were developed to capture the complexity of factors in embedding translational research. Key subthemes identified were education, knowledge, management, time, workplace culture and resources. All thirteen articles identified that a multifactorial approach is required to embed a research culture and translate research findings into clinical practice.

**Conclusions:**

The themes of leadership, organisational culture and capabilities are inherently intertwined and therefore successful strategies require a whole of health approach with organisational leadership driving the strategy, as changing organisational culture takes time and considerable investment. We recommend that public health organisations, senior executives and policy makers consider the findings of this review to provide evidence to initiate organisational changes to support and help create a research environment to drive research translation within the public sector.

## Introduction

Translational research has become a popular approach in recent years as the process aims to bridge the gap between scientific discoveries and practice within health [1]. In the healthcare sector there is a significant time lag of approximately 17 years between the collection of research evidence and its implications into everyday practice [2, 3]. One strategy that has been developed to overcome the time lag is translational research. It is designed to assist in the acceleration of translating research findings into practice [3].

There are varying definitions of translational research [4]. It has been characterised as the application of scientific laboratory discoveries into clinical and non-clinical practice in order to improve health outcomes [5, 6]. Translational research also contains different tiers [4]. For example, in Australia, the NSW Ministry of Health in collaboration with the Cancer Institute of NSW have proposed three tiers [7]. Whilst the number of tiers varies, they all contain the same key features which include pre-clinical trials, clinical trials in which results assist with the formulation of guidelines, plus a focus on implementation and dissemination of research findings [4, 6]. Tier one is the development of laboratory research findings into preclinical studies which are often animal-based; and are essential in understanding how best to develop scientific findings into human trials. Tier two is the testing of new treatments and interventions in humans through clinical trials and aims to facilitate the transfer of knowledge from clinical trials into routine evidence base guidelines. Finally, tier three is the implementation and dissemination of evidence-based guidelines into clinical practice [6, 7].

Barriers to embedding translational research have been reported across an array of disciplines and include inadequate organisational infrastructure, poor research culture, language barriers across scientists and clinicians, and a lack of trained staff who can translate scientific research into clinical guidelines [1, 8]. There is considerable literature looking at barriers and enablers to embedding translational research within academic and research Institutes in the University sector [1], however there is a lack of reports on translational research within public hospital clinical services [9], especially within nursing and allied health professions [10, 11].

A systematic review by Berthelsen emphasises how time factors, lack of authority to change patient care and absence of organisational infrastructure are the most common barriers nurses face in implementing research findings into practice [12]. Also within the nursing profession, it has been reported that there is also a lack of literature discussing the enablers and strategies that are effective in overcoming these reported barriers [11].

Within the allied health profession, there are a considerable number of publications discussing the barriers and enablers to building research capacity and culture but a distinct lack of evidence in how to address the barriers [13]. A lack of financial resources and academic mentorship are recurrent barriers identified by Cordrey and the authors suggest that multidisciplinary research and collaboration are essential factors to help breakdown silos and accelerate the translation of scientific research into evidence-based practice [13].

Given the lack of structured advice, this review aims to investigate, identify, and compare evidence related to the barriers, enablers, and strategies to embedding translational research within a public hospital system, focussing on nursing and allied health disciplines.

## Methods

**Fig 1:**
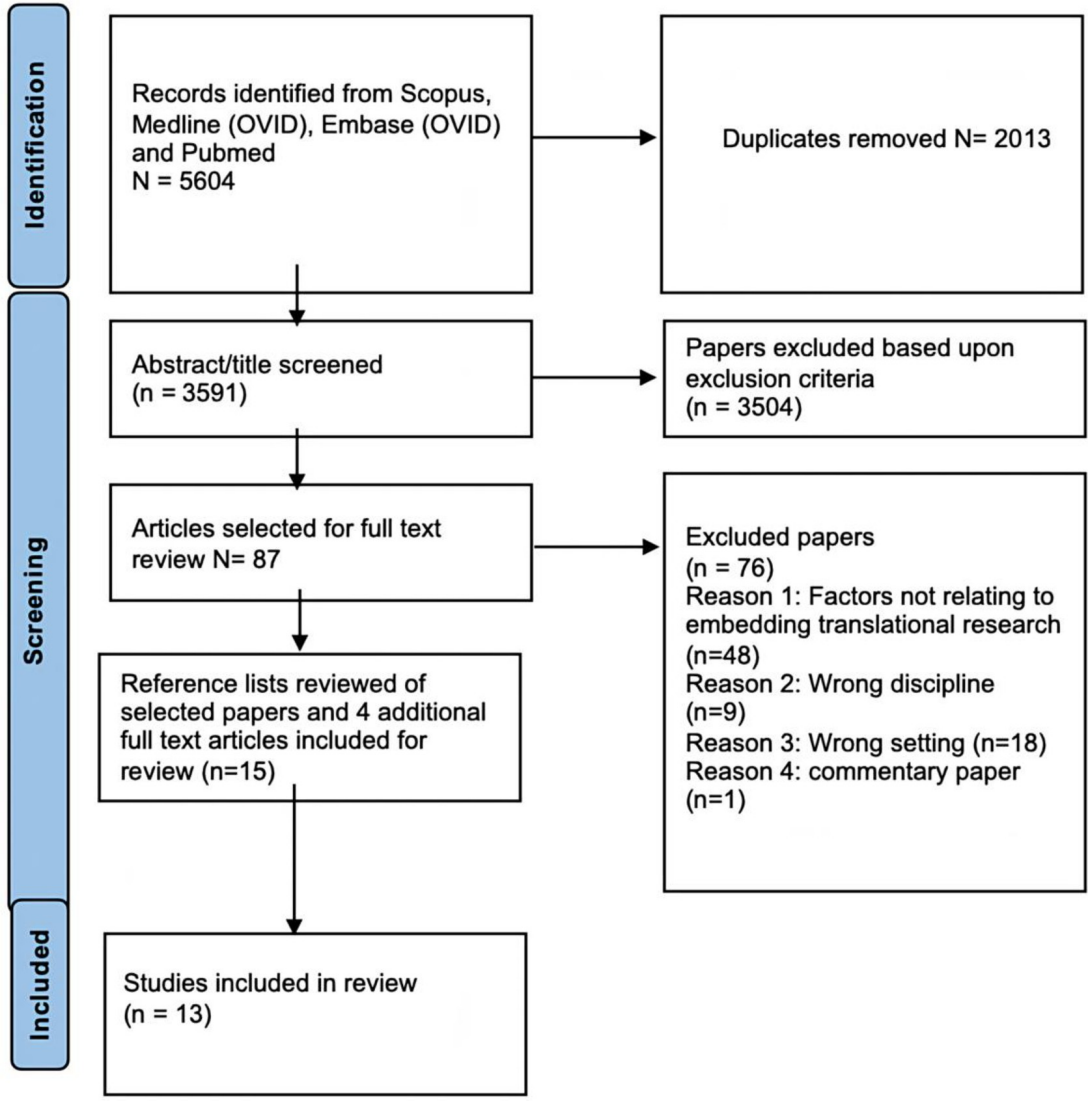
PRISMA flowchart of the literature search and selection process.

This systematic review has channelled the preferred reporting items for systematic reviews and meta-analyses [PRISMA] to ensure the review process follows a structured and approved process [14]. **Fig 1** shows the PRISMA flowchart used for the paper selection process. The review protocol was registered with the Open Science Framework (OSF) in November 2021, (Registration number: 10.17605/OSF.IO/Q29WH).

### Search strategy

**Fig 2:**
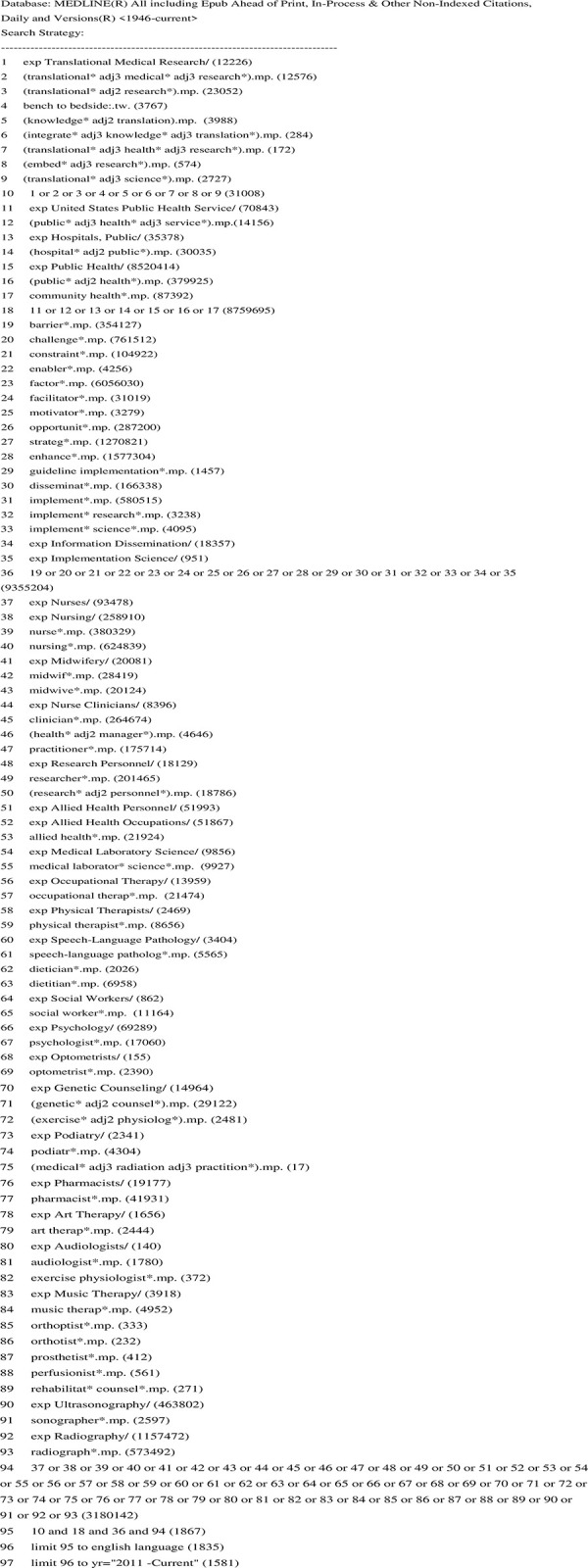
Search strategy example in medline.

The databases searched were Medline (OVID), Scopus, Embase (OVID) and Pubmed and the search strategy used specific search words and combinations, with slightly modified combinations to fit within the structure of each database. **Fig 2** provides an example of the search strategy used for the Medline (OVID) database.

Literature from the last decade related to the barriers, enablers, and strategies in nursing and allied heath disciplines is the focus for this review. **Table 1** outlines the inclusion and exclusion criteria and allied health disciplines were as defined by the Australian Government Department of Health [15].

**Table 1:** Inclusion and exclusion criteria used within the literature search process.

*Characteristics*	*Inclusion Criteria*	*Exclusion Criteria*
*Intervention*	Public hospital, public community health services, tier three translation research, allied health, nursing, midwifery, barriers/enablers/challenges to embedding research, strategies to embed research, guideline implementation, evidence-based practice, dissemination research and implementation research/science, tier two and tier three translational research	Non-human studies, private health service, general practice, private practice, university setting, tier one (pre-clinical studies), all other disciplines, chiropractor, Chinese medicine practitioner, evidence-based practice that focused on patient values and clinical judgement, recruitment outside public hospital
*Study Design*	Randomised control trials, other controlled trials, descriptive and comparative studies.	Systematic reviews, meta-analyses Narrative reviews, editorials, letters, articles, commentary, full-text unavailable
*Publication*	Articles between January 2011 and December 2021 (inclusive)	Studies published prior to 2011
*Language*	English language articles	Non-English language articles

**Fig 3:**
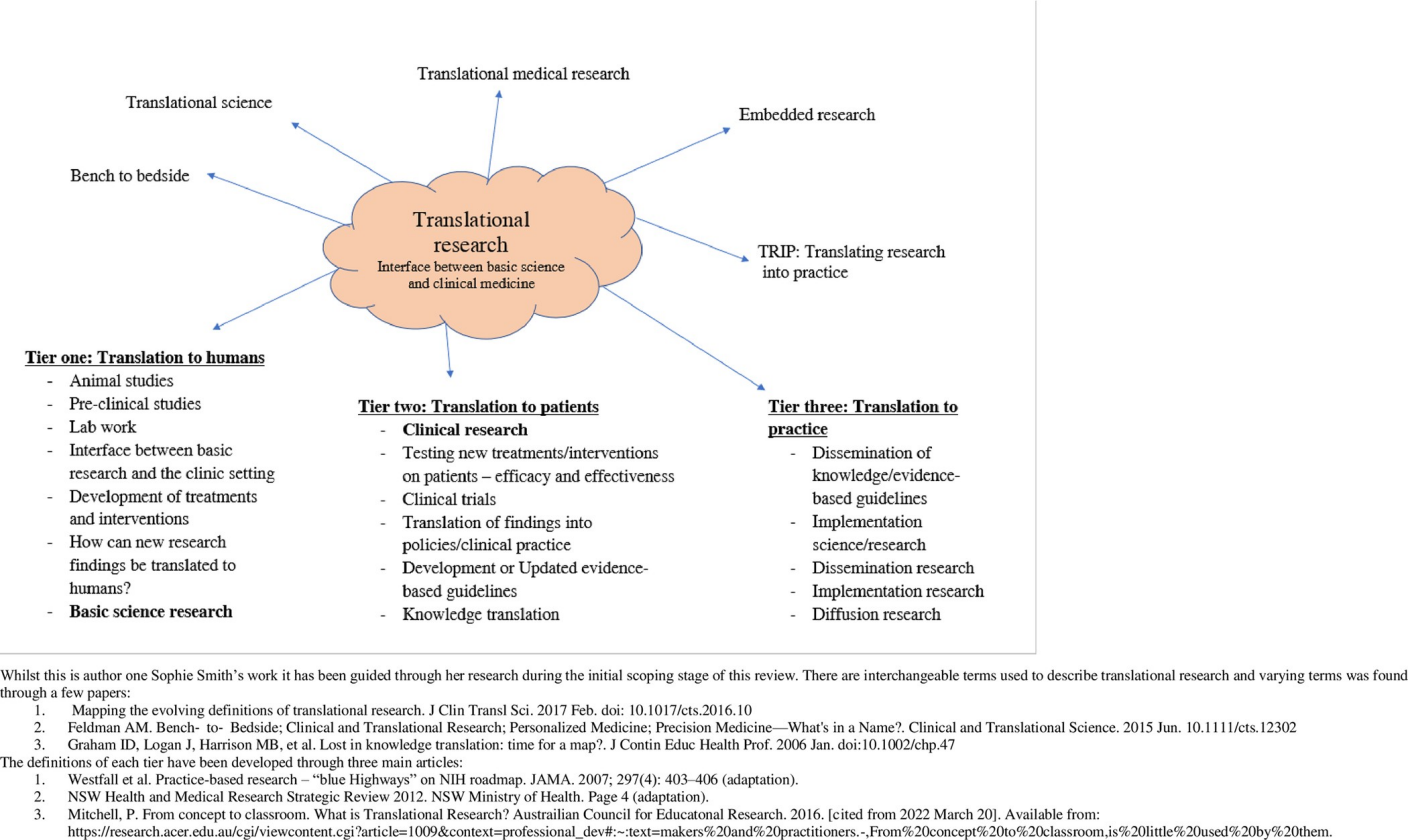
Definition of translational research (authors own work).

The authors adopted the three-tier definition of Translational Research from the NSW Ministry of Health & Cancer Institute NSW model [7]. **Fig 3** was developed to exhibit the terms used interchangeably with translational research and a summary of the specific components encompassing each tier of translational research. During the initial screening of abstracts and titles human studies were considered only, and therefore tier one stage of translational research was excluded as this area frequently involves animal-based studies.

All papers were identified through four databases and imported onto covidence software for screening and extraction. Covidence is a web-based tool designed to assist with the screening and extraction of systematic and scoping reviews [16] and enables asynchronous collaboration between multiple reviewers during the screening process [16]. Duplicates were removed resulting in a total of 3591 papers to be considered for screening. Author one Sophie Smith screened the titles and abstracts against the inclusion and exclusion criteria and 3504 were excluded with the main reasons being that the studies were not based in a public hospital setting or did not include nursing or allied health disciplines. If papers covered other professions such as medicine, to be included the paper must have been centred on nursing and/or allied health disciplines. This led to 87 papers to be included for full text review.

The 87 papers were reviewed in full text by first author Sophie Smith followed by the second reviewer George Johnson. Covidence software was used throughout this process as a means of highlighting any disagreements or discrepancies between the two reviewers. A total of 11 papers were selected. Author one Sophie Smith reviewed the reference list of all 11 papers and included a further 4 papers for full text review. Two papers from the reference lists were included for analysis leading to 13 papers in the final review.

### Data extraction and analysis

Covidence software was used to analyse data contained within each study. Author one (Sophie Smith) through consultation with Author two (George Johnson) modified the data extraction template and the extraction process involved obtaining the study and methodological characteristics, findings, and outcomes from each article. Three extraction sheets were created during this process. The first sheet summarised data extraction findings from each article. A second sheet highlighted the study characteristics across all papers. From this a third sheet was created to identify differences and similarities between the reported outcomes from each article and formed a descriptive analysis of the barriers, enablers, and strategies to embedding translational research across nursing and allied health disciplines. One author Sophie Smith, completed the data extraction process and was audited at random by the second author George Johnson to ensure reliability and validity of the spreadsheets.

### Quality screening process

The review adopted the mixed methods appraisal tool (MMAT) version 2011 [17]. Whilst there is a more recent (2018) version published this did not include a scoring system therefore version 2011 was used to enable both authors Sophie Smith and George Johnson to be able to compare appraisals of each paper [18]. The MMAT inter-reliability has been reported to be moderate-to excellent with a considerable number of systematic reviews using this tool to appraise mixed method studies [17, 19].

This tool consists of a set of items to be answered as yes, no or can’t tell for the following types of mixed methods study/primary studies: qualitative, quantitative randomized controlled trials, quantitative non-randomised, quantitative descriptive and mixed methods [17]. **Table 2** highlights the score of the quality of the papers included in this review. Each paper was scored as either low, moderate, high, or very high. Three papers were scored as low [20–22], one paper as moderate [23], six high [24–29] and three very high [30–32].

**Table 2:** The study characteristics of the included articles in the review and quality score.

*Author*	*Ref no*.	*Study location*	*Aim of study*	*Study design*	*Profession studied*	*Total number of participants*	*Quality of the papers*
Grant et al. [2020]	[24]	Australia	1. Evaluate the organisational context in one Australian adult intensive care setting 2. Evaluate research utilisation in one Australian adult intensive care setting 3. Examine the relationship among dimensions of the context, demographics and research utilisation in this setting	quantitative cross-sectional survey	Nursing	67/205	*** = high
Eames et al. [2018]	[20]	Australia	1. Evaluate the impact of the KT capacity-building program on clinicians KT behaviours within clinical practice 2. Understand the barriers and enablers to clinicians use of KT processes at the outset of the program 3. Identify strategies perceived as useful for supporting their use of KT processes	Quantitative pre/post survey	Nursing	20 completed both pre and post questionnaire, 46 in pre and 39 in post—only data from the 20 were used	* = low
Clement et al. [2016]	[23]	Canada	**1.** To better understand the barriers and facilitators to the use of CCR (Canadian C-spine rule) amongst a wider range of large hospitals and amongst a range of stakeholders, including nurses, physicians and administrators	Qualitative	nurses, physicians and administrators	456	** = moderate
Bennett, Whitehead et al. [2016]	[25]	Australia	1. To describe a participatory action research project for developing this KT capacity building program for OT clinicians. 2. Develop a KT program that would support clinicians understanding and use of the KT process generally as well as the application of this process specifically within the existing organisational context	Qualitative	Occupational therapist	20	*** = high
Bennett et al. [2016]	[32]	Australia	1. To explore the perceptions of occupational therapy staff regarding the influence of these EBP organisational initiatives on workplace culture and clinical practice	qualitative case study	Occupational therapist	30	**** = very high
Aboshiqah et al. [2014]	[31]	Saudia Arabia	1. To explore nurses perception of barriers to research utilisation	Quantitative	Registered nurses	243	**** = very high
Petzold et al. [2014]	[26]	Canada	1. To identify the facilitators and barriers that affect EBP use by OT’s treating individuals with potential acute post stroke USN (unilateral spatial neglect)	Qualitative descriptive	Occupational therapist	9	*** = high
Towell-Barnard et al. [2020]	[27]	Australia	1. Explore and describe the nursing and medical staff perspectives of implementing standardised AT in the ED 2. Explore and describe nursing and medical staff member’s perceptions of standardised AT; their perceived benefits and losses associated with its implementation 3. Identify barriers and enabling factors in the local practice environment to implementation of standardised AT 4. Explore and describe ED nursing and medical staff perceptions of adaptive strategies to facilitate multidisciplinary engagement in implementing standardised AT	Descriptive qualitative	nurses and medical staff	38 nurses and 9 doctors	*** = high
Robinson et al. [2017]	[21]	Australia	1. To explore the engagement of MDT in the WSLHD and NBMLHD in the use and generation of translational research and QI programs. 2. To develop strategies to improve this engagement. 3. Identify key enabling factors for engaging multidisciplinary teams in cancer care across the spectrum of translational research and quality improvement projects	Descriptive qualitative	nurses and medical staff	43 MDT meetings and 18 interviews	* = low
Kristensen et al. [2016]	[28]	Denmark	Elucidate how healthcare professionals in a hospital setting experienced working with the implementation of research results in practice, and which existing methods they utilised to incorporate research results into daily healthcare action	descriptive qualitative	nursing and doctors, manager	12	*** = high
Bayley et al. [2012]	[30]	Canada	1. Seek providers perceptions of the EBR’s in terms of utility and feasibility. 2. Identify barriers and facilitators to implementation of the leg and arm EBR. 3. Observe and record the processes that health care institutions used to implement the EBR’s and determine the uptake of EBR’s.	qualitative	PT, OT, nursing, physicians and hospital managers	79	**** = very high
Wang et al. [2013]	[29]	China	1. Describe the perception of barriers to and facilitators of research utilisation by registered nurses in Sichuan province, China. 2. To explore the factors influencing the perceptions of the barriers to and facilitators of research utilisation.	Quantitative	Registered nurses	521	*** = high
Omer [2011]	[22]	Saudia Arabia	1. Factors that impede or support the introduction of research findings into nursing practice in Saudi Arabia	mixed methods: Qualitative and quantitative	nurses including RN, AIN and nurse manager	413	* = low

Three papers [20–22] were scored low for reasons such as no clear inclusion/exclusion criteria, little to no reflection on the researcher’s potential influence during the observation study and small sample size. Reasons why three papers [30–32] were scored very high included having a clear inclusion/exclusion criterion, use of a validated measurement tool for papers using surveys as their instrument to collect data, a response rate of above 60%, discussion on how findings relate to the context of their study and acknowledgment of potential bias.

## Results

Thirteen studies met our inclusion criteria **Fig 1.** A combination of qualitative [n = 7] [21, 25–28, 30, 32], quantitative [n = 5] [20, 23, 24, 29, 31] and mixed method [n = 1] [22] research designs were represented. **Table 2** provides a summary of the selected studies methodological characteristics. Six articles were from Australia [20, 21, 24, 25, 27, 32], three in Canada [23, 26, 30], two in Saudi Arabia [22, 31], one in China [29] and one in Denmark [28]. Seven [20, 24, 25, 27, 28, 31, 32] of the studies were conducted in a single site hospital setting and the other six [21–23, 26, 29, 30] were across multiple public hospital sites.

Five of the thirteen studies did not report the length of their study [21, 22, 26, 27, 32], however those that did report on the length of their study, the majority [n = 4] were less than 6 months in duration [23, 28, 29, 31]. Two studies [24, 30] were between six to twelve months and two studies were conducted over twelve months [20, 25].

Most of the articles examined barriers, enablers, and strategies on embedding translational research specific to either nursing or an individual allied health profession [n = 12] [20–29, 31, 32]. Only one study discussed barriers and strategies only [30].

Five articles included multiple professions within their study [21, 23, 27, 28, 30]. More nursing profession studies were identified [n = 9] [21–24, 27–31] in comparison to allied health disciplines [n = 5] [20, 25, 26, 30, 32]. Whilst our inclusion criteria covered all allied health disciplines that worked within a public health system, we only identified occupational therapy and physiotherapy professions [20, 25, 26, 30, 32].

A combination of data collection methods was used, which comprised surveys, focus groups, interviews, and observations [20–32]. Surveys were used across seven of the thirteen articles [20, 22–25, 29, 31]. The most commonly reported sample size was less than 100 [n = 9] [20, 21, 24–28, 30, 32] followed by three papers [22, 23, 31] reporting a sample size between 100–500. One paper reported a sample size greater than 500 [29].

### Enablers

**Table 3** provides an overview of the barriers, enablers and strategies reported in the review. The most reported enabler to embedding translational research within the public health system across nursing and allied health professions was leadership, [n = 9] [20–25, 27, 28, 32] which included managerial leadership, mentorship, nursing champions, allied health role models, research active team-leaders and clinical nurse specialists.

**Table 3:** The key barriers, enablers, strategies and overarching themes identified in the included studies.

*Enablers*	*Theme*	*Profession*	*Ref no*.	*Barriers*	*Theme*	*Profession*	*Ref no*.	*Strategies*	*Theme*	*Profession*	*Ref no*.
1. Leadership roles	Leadership/Capabilities	OT, nursing, physio	[20–25, 27, 28, 32]	1. Lack of time	Organisational	OT, nursing, physio	[20–26, 28–32]	1. Leadership roles	Organisational/Capabilities /Leadership	nursing, OT	[20, 22–24, 28, 31]
2. Education	Capabilities/Leadership	OT, nursing	[22, 23, 25–27, 29, 31]	2. Lack of capabilities in interpreting research results	Capabilities	nursing, OT, physio	[20–22, 25, 26, 28, 29, 31]	2. Workplace/ organisational culture [research focused]	Organisational/ Leadership	OT, nursing, physio	[20, 26, 29–32]
3. Access to resources [equipment]	Organisational	OT, nursing	[23, 24, 26, 28, 29, 32]	3. Competing clinical priorities	Organisational	OT, nursing, physio	[20, 23, 25, 27, 28, 30, 32]	3. Education	Organisational/leadership/capabilities	OT, nursing, physio	[22, 23, 25, 26, 29]
4. Motivation	Capabilities/leadership/ Organisational	OT, nursing	[20, 23, 25–28]	4. Lack of authority	Capabilities	nursing, OT, physio	[22, 23, 28–31]	4. Multidisciplinary teams	Organisational	OT, nursing, physio	[20, 24, 25, 29]
5. Management support with research	Organisational/ leadership	OT, nursing	[23, 24, 26, 27]	5. Lack of education/knowledge	Capabilities	nursing, OT, physio	[20, 21, 23, 25, 28]	5. On the job training	Organisational/Capabilities	OT, nursing, physio	[25, 26, 28]
6. Time	Organisational	OT, nursing	[24, 26, 29, 32]	6. Values	Capabilities	OT, nursing	[22, 26, 28, 29, 31]	6. Incentives	Organisational	nursing	[21, 24]
7. Multidisciplinary teams	Organisational	OT, nursing	[23, 26, 27]	7. Lack of leadership	Leadership/Organisational	nursing, OT	[20, 28, 29]	7. Organisational–time	Organisational	OT	[20, 32]
8. Workplace culture	Organisational	OT	[20, 32]	8. Staffing	Organisational	OT, nursing, physio	[25, 30, 32]	8. University affiliation	Organisational	nursing	[21, 24]
9. Experts in the field	Leadership /Capabilities	nursing	[21, 29]	9. Working in silos	Organisational	nursing, OT, physio	[22, 28, 30]	9. Regular guidelines reviews	Organisational	nursing, OT, physio	[29, 30]
10. Financial incentives	Organisational	nursing	[31]	10. Management barriers	Organisation/leadership	nursing, OT, physio	[28–30]	10. Journal club	Organisational/leadership	OT	[20]
11. Student placements	Organisational	OT	[26]	11. Language	Organisational/capabilities	nursing	[22, 29, 31]	11. Access to patient outcome data	Organisational	nursing	[21]
12. Access to patient outcomes	Organisational	nursing	[21]	12. Excessive number of existing guidelines/procedures	Organisational	nursing	[28]				
13. On the job training	Organisational /Capabilities	nursing	[28]	13. Equipment	Organisational	nursing, OT, physio	[30]				

Leadership positions were described [23, 28] as agents of change that provide a supervisory role for other clinicians that assist in the transfer of research findings into clinical practice. Managerial leadership was deemed an enabler through embedding clinical guidelines within a Department to help reduce potential resistance by staff to the uptake of new practices [27]. Managerial support to conduct and utilise research findings was the most common enabler in the papers reporting on the allied health profession. Three papers [20, 26, 32] discussed how managerial support helped create a department that promotes and undertakes research. Management encouraging evidence-based practice, promoting reading of evidence and supporting clinicians in reporting and presenting research findings was deemed important to help build knowledge, dissemination and implementation of research into a department and clinical practice [20, 26, 32]. In particular the influence of clinical nurse specialists in facilitating evidence based-clinical practice [21, 22, 24, 27, 29] was a prominent enabler. Having nursing colleagues with research experience in active leadership roles was reported to support the implementation of evidence-based practices into clinical care [21, 23, 27, 28].

Education was the second most reported enabler [n = 7] [20, 22, 23, 26, 27, 29, 31]. The literature showed a relationship between education and leadership, with the higher qualifications a clinician possessed the more likely they would be in leadership roles and assist with the facilitation of research findings into practice. In addition, further education provided enhanced confidence in evidence-based practice compared to peers with a bachelor degree only [22, 26, 29].

In-services and informal interactions with educators, clinical nurse specialists and medical staff reported a positive correlation with research utilisation within the nursing profession [23, 24, 28, 31], demonstrating the importance of access to educational resources and educators, which was the third most commonly reported enabler [23, 24, 26, 28, 29, 32]. Clinicians who have dedicated education days separate to their clinical days, were identified as more likely to be informed about current research evidence and practices [26].

Motivation was identified in six papers [20, 23, 25–28], as clinicians communicating the benefit of evidence to patients was identified as a driver for stimulating change in clinical practice [27].

Time was also identified as an enabler, with clinicians being provided dedicated time away from clinical duties to focus on reviewing and implementing research [24, 26, 29, 32].

Three papers discussed how multidisciplinary teamwork is important for the promotion of research translation in the public system in nursing and occupational therapy [23, 26, 27]. Each paper discussed the implementation of a new guideline and that working as a team with medical staff, administrators and management facilitates the translation of knowledge across the department and directly into patient care.

Workplace culture was reported as a crucial enabler for knowledge translation in two papers [20, 32] in occupational therapy. Clinicians working in a department that values research and evidence-based practice assists in the dissemination and implementation of translational research. Embedding research active team leaders that are supported by management and the organisation contributes to a research focused workplace culture [32].

Other enablers that were identified were staff having financial incentives to conduct research [31], on the job training in implementing clinical guidelines [28], access to patient outcomes to see the effects of research findings on patient care [21]. Also, student placements can promote research translation through engaging staff to keep up to date with research, as students introduce new evidence-based practices from their university curriculum that health professionals may be unaware of [26].

#### Barriers

Barriers were similar across nursing and allied health with lack of time being the most reported across both disciplines [n = 12] [20–26, 28–32]. Clinical priorities was the third most reported barrier and is directly related to time, as high clinical workload leads to an inability to create time to read research articles or implement evidence-based practices [20, 23–25, 27–30, 32].

Lack of capabilities amongst clinicians was the second most common barrier across eight articles [20–22, 25, 26, 28, 29, 31]. Allied health disciplines highlighted the lack of confidence in their ability to choose which knowledge translation strategies to implement within their department [20, 25]; and within nursing two articles highlighted nurses were incapable of evaluating the quality of evidence within research articles [22, 29].

Education was a key barrier, with seven articles emphasising a lack of evidence-based practice education amongst both allied health and nursing disciplines and a reported unfamiliarity with implementation science/research and knowledge translation strategies [20–22, 25, 26, 28, 29]. Across both disciplines five articles discussed a lack of higher-level post graduate education as a barrier to research translation [20, 21, 23, 28, 32].

Lack of authority was reported in six papers [22, 23, 28–31]. Bayley reported that allied health clinicians did not feel they had the authority to upskill or teach clinicians of other disciplines new guidelines and/or evidence-based practices [30]. Wang, Omer and Aboshaiqah acknowledged through a ‘BARRIER’s scale’ developed to assess the barriers nurses faced, that one of the greatest barriers in nursing implementing translational research into clinical practice, was a feeling of a lack of authority to change patient care [22, 29, 31]. Kristensen reinforces this, as they noted that nurses feel they do not have the authority to use evidence-based practice due to a lack of qualifications [28].

Individual values were a recurrent barrier in five papers [22, 26, 28, 29, 31]. In nursing it was reported that they perceived little benefit in changing practice to patient care based on research findings [22, 29, 31] and a lack of interest in implementing evidence-based knowledge [28]. Within occupational therapy Petzold suggests that some clinicians are set in their ways and not willing to change clinical practice regardless of what the evidence recommends [26]. Individual values on willingness to implement research findings and adopting evidence-base practice are personal barriers that impede the translation of research findings into patient care.

Lack of leadership was reported in four papers within the nursing profession [22, 24, 28, 29]. Grant highlights a lack of formal leadership training in nursing within an intensive care unit which impacts on research utilisation [24]. Kristensen concludes that there are a lack of nurses within research-active leadership positions, which is strongly correlated with the promotion of embedding translational research into patient care [28].

High turnover of both nursing and allied health staff was reported in three papers [25, 30, 32] as a barrier, as there was a lack of direction and support for new staff members to be upskilled in new guidelines, and a difficulty involving rotating staff members in knowledge translational strategies. This is intertwined with management barriers [28–30] with management not being active in enforcing new research into departments, allocating staff resources for implementation processes or financial allocation for equipment required to implement changes.

Three papers [22, 28, 30] identified working in silos was a barrier to the implementation of translational research. Kristensen explores how nurses with research specific roles feel isolated and incapable of making changes on their own [28]. Additionally, nurses acknowledged how a lack of interdisciplinary collaboration between allied health and nursing disciplines made it a significant barrier on implementing evidence-based recommendations to patient treatment plans [30]. Omer discusses the lack of physician cooperation with nurses to implementing research findings into patient care is predominately due to a lack of collaboration between the disciplines [22].

Language barriers was discussed in three papers and only within the nursing profession [22, 29, 31]. This was only reported in papers that were set in either China or Saudi Arabia. Many journal articles are transcribed into English which can impact the dissemination of research findings in these two countries where English usage is not widespread.

#### Strategies

All thirteen articles discussed the complexity of translating research findings into clinical practice and that one strategy alone is not going to address the issue [20–32]. Six articles discussed workplace culture as a key strategy to help involve clinicians in knowledge translation solutions and to create an organisation that is research focused and values evidence-based practice [20, 25, 26, 29, 30, 32]. Creating a research focused workplace culture was reported as starting with management encouraging staff to be involved in research, showing a positive attitude to undertaking projects and supporting staff to present their findings to peers [20, 25, 32]. An example of a workplace culture strategy is senior management using knowledge translation language in staff meetings and communicating with staff about research projects and their findings [25]. A key finding in the literature emphasised the need for all clinicians to be involved in knowledge translation strategies and not just senior leaders or managers. This is to engage clinicians and facilitate knowledge translation within a department [25–27].

Leadership has been described as a powerful enabling strategy to help disseminate research findings across departments and disciplines [20, 21, 23–25, 27–29, 32]. Clinical nurse specialists and educators have been shown to positively influence the encouragement of evidence-based practice through modelling research findings into clinical care that others can follow [20, 21, 23–25, 27–29, 32]. A recurrent strategy identified as a leadership theme is having a dedicated clinician as a knowledge translation champion that can assist in the dissemination and implementation of translational research within the nursing and allied health disciplines [20, 24, 25, 28, 32]. Furthermore, seven papers discussed that having dedicated research positions in Research and Evidence Practice (REP) champions, allied health research champions, research project leaders and research supervisors/mentors such as university academics embedded within the hospital setting can contribute to the diffusion of evidence into practice [20–24, 28, 31].

Education opportunities for staff is an important strategy demonstrated in five papers [22, 23, 25, 26, 29]. A strategy in nursing and occupational therapy is providing educational refreshers on emerging evidence, review of guidelines and evidence-based practice [23, 25, 26]. Omer [22] suggests that a review of the nursing curriculum to increase the level of evidence-based practice and evaluating research skills that is provided in nursing degrees, will improve individual nurses’ capabilities on the utilisation of research into clinical practice. Additionally, Petzold suggests pre-and post-knowledge quizzes to cement new learning and attending research conferences or webinars [26]. Furthermore, Bennett implemented educational outreach and clinical case studies to embed translational research through the department [25].

Enhancing multidisciplinary teamwork was discussed in four papers to improve the translation of research within the public health sector [20, 24, 25, 29]. Bennett implemented a knowledge translation capacity-building program within an occupational therapy department and reported positive outcomes [25]. The program incorporated multifactorial strategies relating to leadership, organisational and capability strategies. They achieved this through focussing on mentorship, clinician engagement, clinical case study reviews, time allocation for research activities, management support, reviewing clinical guidelines and discussing knowledge translation in multidisciplinary team meetings. Finally, the program had a focus on enhancing staff research capabilities through educational outreach and refresher educational sessions.

Organisational strategies reported to address the lack of time for clinicians to embed evidence-base practice activities [20, 32] included, having off-duty clinical time to review guidelines and conduct literature reviews [32], on the job training [25, 26, 28] and incorporating guideline reviews within the workforce for clinicians [29, 30].

University affiliation [21, 24], journal clubs [20] and providing clinicians with incentives to conduct, review and implement research were discussed in two papers[21, 24]. Incentives included funded internships, scholarships for nurses to undertake research studies and secondments for nursing staff to be involved in translational research projects [24, 29].

#### Three overarching themes

In the identification of the enablers, barriers, and strategies to embed research translation, it became clear of the interconnection between the factors. Three overarching themes were developed to capture the key findings of this review and are shown in **Table 3.** The three themes are:

1. ‘Leadership’—research champions, experts in the field, mentors, and supervisors.
2. ‘Capabilities’—skill, knowledge, and values on conducting, understanding, and interpreting research.
3. ‘Organisational Culture’—time, managerial support, departmental culture, and teamwork.

## Discussion

To the best of our knowledge this is the first systematic review aimed at identifying the barriers, enablers, and strategies to embedding translational research within the public hospital system across nursing and allied health disciplines. Three overarching themes were identified namely Leadership, Capabilities and Organisational Culture. These themes and the factors that embed them, as well as considering the selected studies methodological characteristics will be examined.

### Methodological characteristics

There are only five countries that were identified in this review with almost half of the studies conducted in Australia; demonstrating that Australia is showing a strong focus on embedding translational research compared to other countries. Australia, Denmark, Canada, and Saudi Arabia have similarities in their health systems and face similar challenges. To further explore the similarities and differences in these countries that are undertaking translational research studies, we have summarised here their health infrastructure, research funding allocation, population health concerns and how these areas may impact on their output related to translational research.

The Australian health care system is jointly run by Federal, State/Territory and Local government levels [33]. It comprises of public, private and primary health care. The Medicare benefits schedule is the backbone of Australia’s healthcare that enables all Australians to access the public hospital services without cost [33]. This is similar to Canada who have a publicly funded health system called Canadian Medicare [34]. Australia and Canada both offer private health insurance to enable people to access costs of treatment in private hospitals and assist in the coverage of other medical expenses such as dental care.

Denmark has an excellent modern universal care system with all Danish residents entitled to publicly financed care including primary healthcare with no co-payments required for primary health care visits which differs to Australia [35, 36]. Private health insurance is available in Denmark however, only 2.5% of health spending is contributed to private health insurance in comparison to 27.8% in Australia [35, 36]. Whilst Saudi Arabia and China are both classified as developing countries, China’s health care system is vastly different to Saudi Arabia which has a mixed public and private health system, predominately funded through their Ministry of Health, being similar to Australia [37]. China’s healthcare system is a multilevel system with basic medical insurance, commercial health insurance, donations and medical mutual aid activities [38]. China’s multilevel health system is complex and the level and amount of cover provided to citizens depends on location and employment. For employees there is mandatory payments taken from their salary to cover medical insurance [38].

Within these public health structures there is funding allocated to all areas of health including health research. In Australia 15.7% of the total health expenditure was allocated to health research [36]. This enables funding opportunities through for example, the National Health and Medical Research Council (NHMRC) and Medical Research Future Fund [MRFF], two of Australia’s leading funding bodies in health research to drive research capacity and translational research [39]. Canada over the last 20 years have dramatically increased the allocation of funds to enhance health research which is driven by the Canadian Institute of health research [40]. Whilst difficult to obtain the allocation of funding to health research in Denmark the Statens Serum Institute (SSI) is the main research institution in Denmark that centres around public health research with a strong focus on infectious diseases [41].

It is evident that developed countries have greater resources for translational research with a focus on cancer, obesity, cardiovascular disease, diabetes and prevention of communicable disease [42]. Whereas, developing countries are focussed on the elimination of communicable disease, maternal and neonatal death, antimicrobial resistance and climate change as outlined through the World Health Organisation [43]. Furthermore, developing countries such as China as defined by the World Trade Organisation [44] whilst participating in tier one translational research face the challenges of funding security, multidisciplinary collaboration and implementation science that impact the translation of research into health care [45]. Whilst translational research organisations exist within China they lack essential infrastructure such as multidisciplinary teams, government funding and support [46]. To overcome these challenges they have joined forces with Australia in creating an Australia-China joint research centre that assists with increasing research capacity and capabilities through joint partnership [47, 48]. It builds on research opportunities and embedding research and innovation within the Chinese and Australian healthcare systems.

Whilst there are differing challenges faced between developed and developing countries it is evident that creating international partnerships such as the Australia-China joint research centre can help drive research capacity and embed research and assist in overcoming lack of resources that many developing countries encounter that can impact the delivery of translational research.

Whilst five papers did not disclose the length of their studies [21, 22, 26, 27, 32], the majority were conducted over less than six months. The two papers [20, 32] that were conducted over 18 months highlighted that more time was warranted to see if the strategies contributed to change in practice over a sustained period of time, and not just during the immediate implementation phase. It may be beneficial to conduct future studies over a longer period to enable researchers to understand if change is sustained, and if ongoing interventions are required to perpetuate positive outcomes.

Nine papers [20, 24–30, 32] had less than a hundred participants with Petzold and Kristensen using interviews and focus group methods with less than 20 participants [26, 28]. Interviews and focus groups are advantageous as they can obtain rich exploratory data [49], however the smaller sample sizes may impact the generalisability of the findings. Additional studies using alternate data methods with a larger sample size would have provided a more representative generalisable sample. In addition, only two papers [21, 30] used observations as a study method. Ethnography observational studies may enable researchers to understand the culture of the healthcare team and learn insights into the barriers and enablers of embedding translational research within a public healthcare setting that interviews may not identify [50]. In addition, observational research may be more practical for evaluating longitudinal programs, as they are more adept at showing changes over time [51]. A scoping review conducted by Gertner explored the benefit of ethnographic approaches in implementation science and concluded that it is a sound method in understanding complex interactions in embedding research findings into the healthcare setting [52].

### Leadership

This review identified the importance of clinical leadership in helping to embed research implementation. Fitzsimons and Cooper [53] support the importance of leadership, as they reported that clinical nurse specialists who have additional training and experience in evidence-based practice led to a positive correlation on the dissemination of guidelines and research findings to other colleagues. Kristensen states that clinical nurse specialists are agents of change providing expertise, mentorship, and supervision to other nurses [28]. Towell-Barnard reported that senior staff who had higher qualifications and research experience were perceived as change leaders and had a positive impact on the implementation of evidence-based guidelines within an emergency department [27].

Whilst leadership was identified across both disciplines in this review, it was more prominent in the nursing literature as an enabler for embedding translational research into practice [21, 23, 24]. However, Eames highlighted that one of the most useful strategies is embedding allied health research positions and the creation of knowledge translation champions within a department; as these positions enable the upskilling of other clinicians through mentoring, supervision and role-modelling changes in clinical practice [20]. This is a similar finding identified through the qualitative evaluation by Wenke and Ward that demonstrated allied health research positions increase evidence-based practice, enhance research infrastructure and improve workplace culture and professional development [54].

The impact of services and initiatives lacking effective leadership was identified in this review, and this is supported by Robinson who reported that there are very few nurses involved in research projects [21]. Currey, Considine and Khaw recommend the creation of clinical nurse research consultant leadership positions due to the lack of highly specialised clinical research nurses in Australia [55]. Kristensen states that research-led positions are ideally to be held by nurses who have a doctorate [28].

Managerial support which is interspersed between both the leadership and organisational themes developed in this review, and acts as an important influence in increasing capabilities of staff in evidence-based practice. Unfortunately, managerial barriers such as lack of support for research training and not prioritising tier three translational research was evident in three papers [28–30]. This is reinforced in a recent systematic review by King concluding that organisational and leadership support is pivotal for research utilisation amongst nursing and allied health disciplines, but a lack of managerial support has been a considerable barrier affecting research translation [56]. Managerial support can also be in the form of financial incentives and promoting the attendance of conferences or undertaking short courses [26]. Managers can also implement strategies to demonstrate the value of evidence-based practice, through providing patient data outcomes and sharing this data with colleagues [21].

### Capabilities

Both nursing and allied health disciplines emphasised a lack of evidence-based practice skills and trouble interpreting research findings, resulting in a failure to implement them into clinical practice [20, 22, 25, 26, 29, 31]. Borkowski’s systematic review highlighted that in all allied health professions there was a lack of research skills and confidence in their abilities to interpret and analyse research findings [57]. Additionally, during the implementation phase nurses did not feel they had the authority to make changes to clinical care [22, 29]. Petzold and Kristensen perceived on the job training embedded within nursing and allied health departments to be an efficient way to implement research findings into clinical care [26, 28].

This review has identified that education is a complex and layered factor in embedding translational research, impacting on research knowledge, leadership position, communication, prioritisation of research training, and staff motivation to implement evidence-based practice. It is crucial to employ strategies that increase education amongst nursing, and evidence has indicated that the nursing curriculum at universities should be reviewed to provide opportunities to enhance research skills [56, 58]. Nurses need research knowledge and education to feel empowered to be able to embed translational research findings into the care of their patients and services [59]. There is limited evidence analysing strategies to assist in empowering nurses to be part of the decision-making process in clinical care [59], and therefore it is important to implement and evaluate educational initiatives and their impact on clinicians confidence to use, interpret and apply research evidence in their service setting.

### Organisational culture

An organisational culture should be focussed on promoting evidence-based practice, research educated and motivated leadership, and enhancing staff capabilities to undertake and understand research, which are all crucial to embedding research translation in the public system. A culture focused on supporting research and evidence-based practice will promote leaders and managers with the skills and capabilities to drive research translation across the organisation [60, 61]. The importance of organisational culture is reflected in this review, as it is the most recurrent theme across our enablers, barriers, and strategies.

Time was a significant barrier for both allied health and nursing professions. Competing clinical priorities and staff shortages are key factors playing a role in a lack of time for clinicians to review literature, guidelines and be involved in research projects [20, 23, 25, 27, 28, 30, 32]. Whilst managerial and organisational culture has been noted as crucial in overcoming time as a barrier for embedding translational research into the public health system, further research into evaluating appropriate strategies to address this barrier is recommended [20, 32]. The health system is a demanding environment and constraints related to a lack of time and resources are a common concern for departments and managers that cannot easily be addressed. However, reviewing staff resources, organisational prioritisation and initiatives to promote research practices and embed a research culture within the organisation, is crucial to make sustained change to support evidence-based practice.

Siloing of disciplines is another issue that was identified when nurses and allied health staff are attempting to implement guidelines and evidence into practice [30]. Organisations promoting increased cross-disciplinary teams, working together in developing evidence based clinical guidelines, have shown increasing understanding across teams and a collaborative inclusive approach, that enhances skills across services [62]. Urquhart recommends a transdisciplinary approach, whereby “different disciplines work together to develop and use a shared conceptual framework that integrates discipline-specific concepts to address a common problem” [63, p 2.]. Laschinger supports a multidisciplinary approach and discussed that to empower nurses they need to be included in medical teams where decisions on patient care are made to create a shared governance within the public health system [64]. Multidisciplinary or transdisciplinary approaches are an important enabler as they enhance communication, create shared empowerment, adherence to guidelines and clinical decision making [21]. Rather than focusing on strategies for individual disciplines, it is important to create a collaborative team environment that engages multiple disciplines, to assist in breaking down silos and creating a united and empowered workforce [20]. Robinson recommends multidisciplinary teams implement strategies including: access to patient data outcomes, regular research forums and embedded research fellowships to drive tier one and tier two translational research [21].

Motivation is an important enabler to the implementation of research into clinical care [20, 23, 25–28] and is interlinked with our themes of organisational culture and leadership. An organisation that promotes evidence based practice, creates opportunities that encourages staff to work on research projects, provides time away from clinical priorities and develops leaders that promote these practices, has a direct effect on an individual’s value and motivation to implement research into their clinical care [60].

## Study limitations and strengths

Whilst all public hospital allied health professions were included in the search strategy only physiotherapy and occupational therapy disciplines were included in the final review. Therefore, this review is not generalisable to other allied health professions, however, it has highlighted the lack of evidence in allied health and a need to focus future research on these disciplines.

This review completed a large search strategy that included all relevant allied health professions, interchangeable terms used to define translational research and synonyms of barriers and enablers to assist in identifying papers. Another strength of this review is that we used the PRISMA guidelines to structure this systematic review which optimises the quality of our reporting.

Only Medline, Embase, Scopus and PubMed databases were searched as they were deemed the most appropriate databases through an initial review of literature to determine the most appropriate databases to include. Therefore, some publications may have been missed if not identified in the four databases. Similarly, this review only included studies in English language, plus grey literature and unpublished studies were not included in our search strategy which may impact the comprehensiveness of this review. However, references of all included studies were analysed for inclusion to reduce the likelihood of relevant papers being missed.

### Implications & recommendations

The themes identified in this review are inherently intertwined, and successful strategies require an organisational approach from the top down to drive a research focussed culture, to support the development of capable and research educated clinical and managerial leadership, through strong managerial support and education provision, in order to embed evidence-based practice at the departmental level.

Clinical nurse specialists, managers and research leaders have a crucial role in providing expertise, mentorship and supervision, and it is important they are educated in research practice, research active and familiar with knowledge translation. Knowledge translation needs to be embedded in daily practice, through managers incorporating it in meetings and providing informal research evidence discussions and journal clubs. In addition, clinicians need support through dedicated education days, backfilling of staff, providing free research education, and promoting research and academic seminars to discuss current research findings.

In addition, this review has highlighted that nurses and allied health staff are not receiving enough training in research, and modifying their degree curriculum content should be considered to include additional research projects to enhance familiarity and confidence in research practice. Through enhanced education and qualifications in research, this will empower clinicians to ensure they have the authority to develop and discuss clinical guidelines and review evidence-based practices.

Successful initiatives require a multifactorial approach facilitating multidisciplinary teams that utilise modern research and patient reported outcomes, to develop clinical priorities focussed on patient centred care. Given the complexity and time required to modify an organisations culture, it is recommended future research consider sustained interventions with longitudinal follow up to assess impact over time.

A program that captures many of the identified strategies in this review, is Bennett knowledge translation capacity-building program for occupational therapists [25]. The program used a multifactorial, multidisciplinary approach and applied leadership, organisational and capacity building strategies including, mentorship, provision of time, management and education support. Eames evaluated the program and reported improvements in clinicians understanding and confidence of translational research; and that the program exhibited a change in the department’s culture in engaging all staff in knowledge translation activities [20]. This review recommends that nursing adopts and adapts Bennett’s program and considers strategies reported in this review to modify the program to ensure it is aligned with the current challenges faced by nursing [25]. For allied health, we encourage increased research across the broad discipline to address the lack of literature identified in this review.

## Conclusion

This review has provided a detailed overview of the challenges nursing and allied health disciplines face in embedding translational research into clinical practice. The review has outlined strategies and approaches to overcome the challenges and identified three key overarching themes namely: leadership, organisational culture and capabilities.

Successful strategies require a whole of health organisational approach, strong research focussed leadership and a multifactorial and multidisciplinary plan to effectively impact the culture, disciplines and departments over time. Strategies need to break down silos, create enhanced communication, promote shared empowerment, support research and education and adhere to clinical guidelines, to drive evidence-base practice within nursing and allied health.

We recommend that public health organisations, senior executives and policy makers consider the findings of this review to provide evidence to initiate organisational changes to support and help create a research environment to drive research translation within the public sector.

## Supporting information

S1 Checklist PRISMA 2020 checklist. (DOCX)
